# Do Interventions Designed to Support Shared Decision-Making Reduce Health Inequalities? A Systematic Review and Meta-Analysis

**DOI:** 10.1371/journal.pone.0094670

**Published:** 2014-04-15

**Authors:** Marie-Anne Durand, Lewis Carpenter, Hayley Dolan, Paulina Bravo, Mala Mann, Frances Bunn, Glyn Elwyn

**Affiliations:** 1 Centre for Lifespan and Chronic Illness Research, University of Hertfordshire, Hatfield, United Kingdom; 2 School of Nursing, Pontificia Universidad Catolica de Chile, Santiago, Chile; 3 Support Unit for Research Evidence, Cardiff University, Cardiff, United Kingdom; 4 Centre for Research in Primary and Community Care, University of Hertfordshire, Hatfield, United Kingdom; 5 The Dartmouth Center for Health Care Delivery Science, Dartmouth College, Hanover, United States of America; Universidad Peruana Cayetano Heredia, Peru

## Abstract

**Background:**

Increasing patient engagement in healthcare has become a health policy priority. However, there has been concern that promoting supported shared decision-making could increase health inequalities.

**Objective:**

To evaluate the impact of SDM interventions on disadvantaged groups and health inequalities.

**Design:**

Systematic review and meta-analysis of randomised controlled trials and observational studies.

**Data Sources:**

CINAHL, the Cochrane Register of Controlled Trials, the Cochrane Database of Systematic Reviews, EMBASE, HMIC, MEDLINE, the NHS Economic Evaluation Database, Open SIGLE, PsycINFO and Web of Knowledge were searched from inception until June 2012.

**Study Eligibility Criteria:**

We included all studies, without language restriction, that met the following two criteria: (1) assess the effect of shared decision-making interventions on disadvantaged groups and/or health inequalities, (2) include at least 50% of people from disadvantaged groups, except if a separate analysis was conducted for this group.

**Results:**

We included 19 studies and pooled 10 in a meta-analysis. The meta-analyses showed a moderate positive effect of shared decision-making interventions on disadvantaged patients. The narrative synthesis suggested that, overall, SDM interventions increased knowledge, informed choice, participation in decision-making, decision self-efficacy, preference for collaborative decision making and reduced decisional conflict among disadvantaged patients. Further, 7 out of 19 studies compared the intervention's effect between high and low literacy groups. Overall, SDM interventions seemed to benefit disadvantaged groups (e.g. lower literacy) more than those with higher literacy, education and socioeconomic status. Interventions that were tailored to disadvantaged groups' needs appeared most effective.

**Conclusion:**

Results indicate that shared decision-making interventions significantly improve outcomes for disadvantaged patients. According to the narrative synthesis, SDM interventions may be more beneficial to disadvantaged groups than higher literacy/socioeconomic status patients. However, given the small sample sizes and variety in the intervention types, study design and quality, those findings should be interpreted with caution.

## Introduction

Increasing patient engagement in healthcare is now considered one of the goals of medicine and a priority on the policy agenda [1], [2]. Shared decision-making (SDM) is one of the consultation models advocated to promote patient activation and engagement in healthcare [3], [4]. It offers a new paradigm to manage patients' growing demand for healthcare by promoting collaborative decision-making between patients and clinical experts. However, there is a risk that SDM primarily attracts and benefits those who are natural information-seekers, who are educated, empowered and able to advocate for their needs, while marginalising patients who are socially excluded and disadvantaged [5]. The idea has therefore emerged that SDM may increase health inequalities. Research shows that involving patients in their care and listening to their views improves knowledge, decision outcomes, compliance with treatments, and reduces the uptake of elective procedures [6]. However, engaging in SDM generally requires knowledge, confidence, self-efficacy and high levels of health literacy.

Decision Support Interventions, also known as Patient Decision Aids, have been developed to help patients clarify their values and preferences while providing evidence-based information about the options' harms, benefits and outcome probabilities [7]–[9]. Existing interventions and SDM programmes are primarily web-based, information-rich and sophisticated. They are likely to give a differential advantage to patients who are computer and health literate, already educated and socioeconomically advantaged, and risk marginalising those who are underserved and disengaged, therefore increasing health inequalities [10]. The benefits of decision support interventions have been demonstrated in a meta-analysis of over 80 randomised controlled trials [6]. However, their impact on disadvantaged groups, who concurrently experience the highest burden of disease, have never been investigated in a systematic manner. The Cochrane review of decision aids for people facing health treatment or screening decisions called for more research on the impact and accessibility of decision support interventions in underprivileged populations [6].

People from disadvantaged groups, and particularly those with low literacy, represent a large proportion of the population in most developed countries. It is estimated that 80 million Americans have limited heath literacy [11]–[13]. They are burdened by worse health outcomes than more literate and educated groups, and have limited access to care [11]. Evidence suggests that younger patients, women and people from higher socioeconomic groups are more likely to assume an active role in medical decision-making [14], [15]. All patients can be vulnerable to the power imbalance of the patient-provider relationship [16], but those who experience lower literacy, lower self-efficacy and a higher burden of disease are likely to be at a greater risk of marginalisation, poor disease management, and worse health outcomes. This systematic review aims to assess the impact of SDM interventions on patients from disadvantaged groups, and on health inequalities.

## Methods

A protocol was developed in advance to outline the objective and methods of the systematic review. It was registered in Prospero in March 2012 (Registration number CRD42012002200). We planned and reported the systematic review in accordance with the preferred reporting items for systematic reviews and meta-analyses (PRISMA)(see Checklist S1) [17].

The following research questions were used to guide the systematic review process:

- Can SDM interventions improve outcomes for disadvantaged groups?
- Can SDM and related interventions decrease health inequalities?
- What are the features of SDM interventions that are beneficial to disadvantaged groups and influence health inequalities?

### 2.1. Search strategy

The search strategy was developed with an Information Specialist (MM) and piloted in OVID MEDLINE (see supplemental file). The following electronic databases were searched from inception until June 2012: CINAHL, Cochrane Central Register of Controlled Trials, Cochrane Database of Systematic Reviews, EMBASE, HMIC, MEDLINE, MEDLINE In-Process and Other Non-Indexed Citations, NHSEED, Open SIGLE, PsycINFO and Web of Knowledge. Conference proceedings and the reference list of all primary and review articles were hand searched. A “cited by” search and “related articles” search was also performed on PubMed. We used social media to contact 378 experts in the area of SDM and patient-centred care. Details of the search strategy are available in appendix 1 as part of our supplementary information.

### 2.2. Study selection and inclusion criteria

We included all studies that met the following criteria: (1) assess the effect of SDM interventions on disadvantaged groups and/or health inequalities, (2) include at least 50% of people from disadvantaged groups, except if a separate analysis was conducted for this group. There were no language restrictions.

A disadvantaged group was defined as all people who are socially disadvantaged in respect of: 1) poverty/socioeconomic status; 2) ethnic minority status; 3) education/literacy level or 4) geographical location (areas described as disadvantaged/or medically underserved), using the author set criteria. Studies of psychiatric patients were included. All conditions and clinical settings (e.g. lay care, primary care, secondary/tertiary care) were included.

SDM interventions were defined as all interventions or strategies designed to engage disadvantaged patients in medical decision-making and/or facilitate SDM, patient involvement in medical decision-making and patient activation. This includes physician coaching, patient coaching, skills workshops and patient prompts, provided the aim is to increase patient engagement in SDM. Educational or self-management interventions that exclusively targeted knowledge were excluded from the review. However, we included educational or self-management interventions that targeted activation and involvement in medical decision making, as well as knowledge.

Three researchers independently screened the title and abstract of retrieved records (M-A D, HD, PB), and the full-text articles meeting the inclusion criteria. Disagreements were resolved by discussion.

### 2.3. Data extraction and quality assessment

Independent double data extraction was performed (M-A D, HD, PB) using a pre-designed form adapted from an earlier systematic review [18], and piloted prior to data extraction. We extracted information about the 1) the author(s)/publication year, 2) type of publication 3) country, 4) source of funding, 5) aims, 6) duration, 7) study type, 8) methodological approach, 9) recruitment procedure, 10) theoretical framework, 11) participant characteristics and sample size, 12) setting, 13) type of intervention, 14) duration of intervention, 15) follow-up, 16) control condition, 17) methods of analysis, 18) number of participants enrolled, included in analysis, withdrawn and lost to follow-up for both intervention and control groups, 19) outcome measures.

Independent dual rating was performed to consider and appraise the quality of included studies. Inconsistencies were resolved through moderated discussions. The quality of randomised trials was rated against the Cochrane risk of bias tool [19]. Observational studies were assessed against Downs & Black quality assessment checklist [20].

### 2.4. Evidence synthesis

Results of all independent groups and repeated measures studies, for which data was available, were combined using the methods described below. In studies where there were more than two groups, we included the groups that were closest to a control and SDM intervention groups. For example, in Cooper et al.'s studies, which used a two-by-two factorial design, we chose the group known as ‘reference’ group in the study as control group, against the ‘physician minimal and patient intensive’ group, which best mirrored the typical features of an SDM intervention, which are primarily targeted at patients. For the purpose of the meta-analysis, we included all quantitative outcomes that were directly relevant to SDM. We were forced to exclude results relating to the acceptability of the intervention, which had primarily been measured qualitatively. However, these results were considered in the narrative analysis. If there were several interventions being evaluated, only shared decision-making interventions (as opposed to an information leaflet) primarily targeted at disadvantaged patients were included in the meta-analysis.

For studies that reported outcome measures with continuous data, standardised mean difference (SMD) was used to calculate effect sizes. Two types of studies were identified, independent groups and repeated measures. For independent group designs, the SMD was calculated using the Hedges' g method [21]. For repeated measures design, Glass's Δ method was used to calculate the effect size [22]. It has been shown that the variance of repeated measures can often be over-estimated and was therefore adjusted using the methods described by Becker [23].

For studies that reported outcome measures as a proportion, odds ratios were used to calculate the effect size. A proportion of patients with a correct and incorrect answer on questionnaires were presented for both pre and post intervention or control and intervention groups. These were used to calculate the odds ratio. Where a questionnaire included multiple questions, an average was taken to leave just one effect size. Log odds ratio and standard error were entered into the meta-analysis.

A random-effects model was used to estimate the weighted treatment effect, including 95% confidence intervals for each outcome measure. The I^2^ statistic was reported to indicate the level of heterogeneity within the effect estimates. Meta-regression was used to investigate the effect of covariates on the overall effect estimates, and where numbers where feasible a stratified analysis was also undertaken. Funnel plots were used to investigate the potential publication bias of the studies included in the meta-analysis. Significance was assumed at P<0.05. All analyses were undertaken using Stata (version 11).

A narrative synthesis was conducted in parallel.

## Results

### 3.1. Description of studies

**Figure 1:**
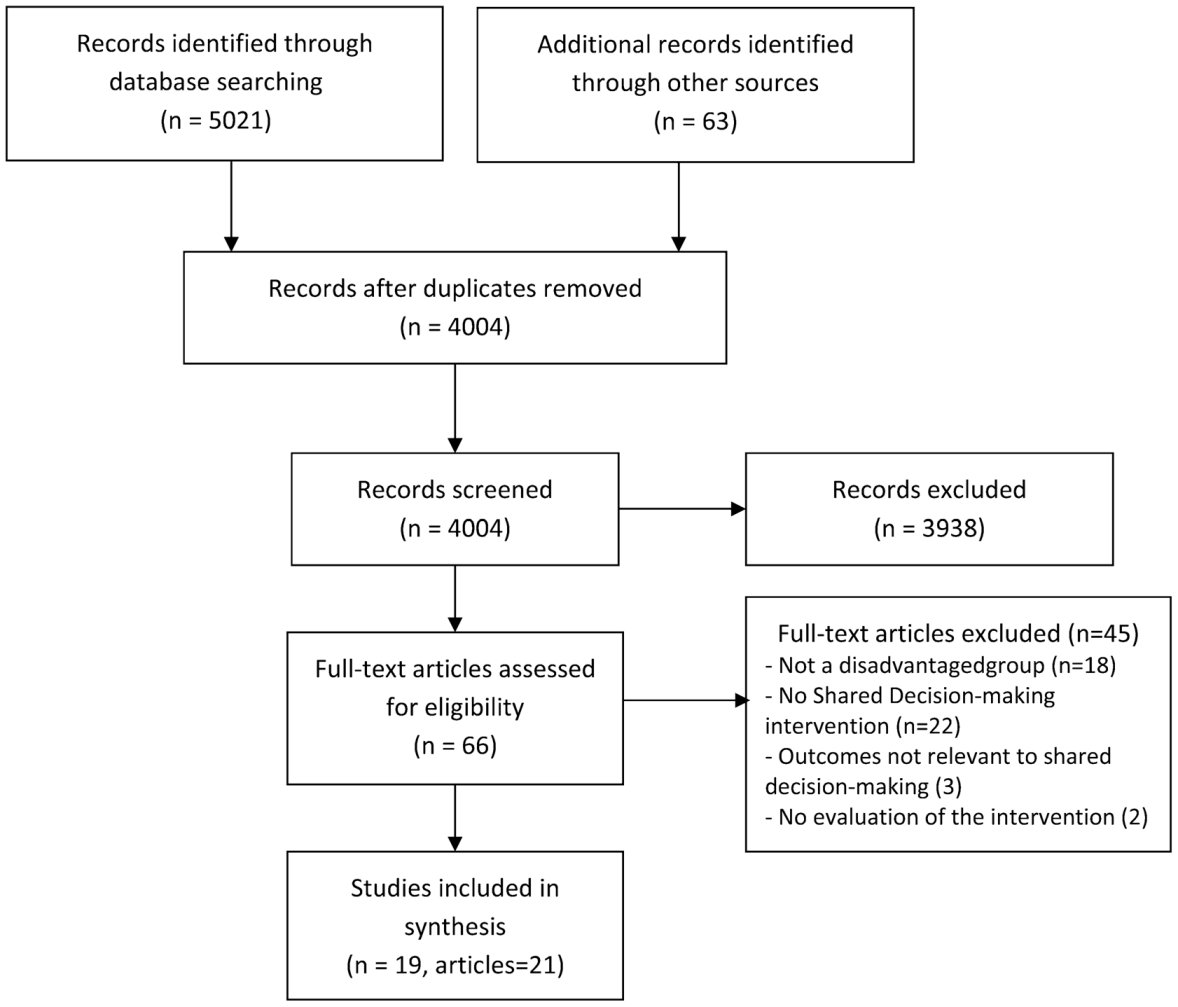
Prisma flow diagram.

Nineteen studies, reported in 21 published articles, met our inclusion criteria (see Figure 1). They presented data collected in primary care, secondary/tertiary care and community settings, in three countries (USA, Australia, Nicaragua) with 84% of studies undertaken in the USA (see Table1). We note that 53% of all studies included were published in the past two years. The total number of participants across all included studies was 4505.

**Table 1:** Characteristics of included studies.

Study	Design	Participants	N	Intervention	Outcomes	Quality score
Bylund 2011	Before/after pilot study	Minority ethnic group (African American: 40.6%; Asian: 40.6%; Hispanic: 12.5%: White: 6.2%)	32	Communication skills workshop.	Anxiety; Patient report of communication behaviour; Patient intent of future use of communication skills; Acceptability of the intervention.	12/26
Cooper 2011*	RCT	Low socioeconomic status and minority ethnic group (African American: 62%, Asian: 1%, American Indian: 0.7%, White: 36% and 76% unemployed)	279	Pre-consultation coaching delivered by trained community health workers focussing on engagement, activation and empowerment skills.	Physician communication behaviours; Patient perceived involvement in care and patient ratings of physicians' participatory decision-making; Blood pressure; Self-reported medication adherence.	Cochrane risk of Bias assessment (see Figure 2)
Drake 2010*	Quasi-experimental	Minority ethnic group (African American:100%)	73	Small group education sessions about PSA testing designed to promote knowledge and self-efficacy among African American men.	Knowledge; Decisional conflict scale; Decision self-efficacy; Preference for control.	14/26
Driscoll 2008*	Observational study	Low socioeconomic status compared to high socioeconomic status and minority ethnic groups	361	Two interventions: 1) About PSA testing only 2) About other health issues and screening for colon cancer.	Knowledge; Discussion about PSA test with physician; Intention to have the PSA test.	5/26
Jibaja-Weiss 2006*	Before/after pilot study	Low literacy minority ethnic group (African American: 29%, Hispanic = 55%, white = 16%)	51	A computerised decision aid about breast cancer surgery, offering both entertainment education and factual information.	Knowledge; Decisional conflict (low literacy version); Acceptability and use of the intervention.	8/26
Jibaja-Weiss 2011	RCT	Low literacy minority ethnic group (exact breakdown unknown)	100	A computerised decision aid about breast cancer surgery, offering both entertainment education and factual information.	Knowledge; Decisional conflict (low literacy version); Surgical treatment preference; Satisfaction with surgical decision; Satisfaction with decision-making process.	Cochrane risk of Bias assessment (see Figure 2)
Kakkilaya 2011	Pilot RCT	Low socioeconomic status (+ low literacy minority ethnic group) (exact breakdown unknown). 69% had income of less than $10,000	89	Prenatal standardised counselling with visual aid.	Knowledge; Attitude/preferences towards resuscitation. Literacy (REALM)	Cochrane risk of Bias assessment (see Figure 2)
Kim 2001	Observational study	Minority ethnic group (White = 50%, African American = 43.3%, Asian American = 6.7%. 36.7% of participants had lower than 9th grade literacy level	30	CD-ROM decision aid for prostate cancer including videos.	Knowledge; Level of satisfaction; Treatment preferences; Treatment intention; Literacy (REALM).	11/26
Kim 2003	Experimental design with randomised control groups	Medically underserved patients (recruited in Indonesia)	768	‘Smart patient’: individual coaching on family planning issues to promote patient involvement in the consultation.	Participation in the consultation; Active patient communication (expressing concerns, asking questions, seeking clarification).	Cochrane risk of Bias assessment (see Figure 2)
Kim 2007	Observational study	Medically underserved and low education (recruited in Nicaragua)	426	Physician coaching on family planning issues.	OPTION tool to measure SDM in the consultation.	12/26
Kripalani 2007*	RCT	Low literacy minority ethnic group (African-American = 90.4%, Caucasian = 8%, other = 1.6% and 78.8% reading level below 9th grade)	250	Two low-literacy paper-based interventions: one promoting informed decision-making about prostate cancer screening and the other intervention encouraging patients to discuss prostate cancer with their doctor.	PSA discussion; PSA test ordered; Literacy (REALM).	Cochrane risk of Bias assessment (see Figure 2)
Miller 2011	RCT	Low literacy minority ethnic group (56% had limited literacy)	264	A web-based decision aid for colorectal cancer specifically tailored for low-literacy patients.	Reported test preference; Readiness to receive screening; Ordered screening after visits; Completed screening; Literacy (REALM).	Cochrane risk of Bias assessment (see Figure 2)
Ross 2010	Quasi-experimental	Minority ethnic group (African American = 100%, 39% had limited literacy)	49	Evidence-based video intervention promoting informed decision-making about PSA screening, targeted at African American men with different literacy levels.	Health literacy (TOFHLA); Knowledge; Accessibility/acceptability of the intervention.	10/26
Rovner 2004	Quasi-experimental	Non college educated minority ethnic group (black = 48%, non-college educated = 43%) Separate analysis for that group	188	Educational videotape about Benign Prostatic Hyperplasia (BPH) and its treatment.	Health literacy (S–TOFHLA); Knowledge; Attitude towards SDM; Readiness to decide.	12/26
Smith 2010*	RCT	Low socioeconomic status	572	Booklet and DVD Decision aid for bowel cancer screening designed for low literacy and low education patients.	Informed choice; Knowledge; Screening attitudes and behaviour; Involvement preferences; Decisional conflict (low literacy version); Decision satisfaction; Confidence in decision-making; General anxiety; Interest in screening; Worry about developing bowel cancer; Acceptability of materials.	Cochrane risk of Bias assessment (see Figure 2)
Trevena 2008*	RCT	Low socioeconomic status and low education (53% left school at age 16 or less)	314	Decision aid booklet about outcomes of biennial faecal occult blood testing screening.	Informed choice; Integrated decision; Knowledge; Clear values; Intention to screen; Test uptake; Short-form state anxiety scale; Decisional conflict; State anxiety; Decisional conflict; Acceptability of the intervention.	Cochrane risk of Bias assessment (see Figure 2)
Volandes 2010*	Observational study	Minority ethnic group with marginal literacy (African American = 56%, white 44%)	146	A 2-min video of a patient with advanced dementia.	Literacy (REALM); Uncertainty subscale of the decisional conflict scale; Preference for aggressive care.	16/26
Volk 2008*	RCT	Low literacy and high literacy groups	450	Entertainment-based patient decision aid for prostate cancer screening and audio-booklet.	Acceptability of the intervention; Engagement with the intervention; Knowledge; Decisional conflict scale; Self-advocacy scale.	Cochrane risk of Bias assessment (see Figure 2)
Wray 2011*	Observational study	Minority ethnic group (African American = 100%)	63	Educational outreach strategy promoting informed decision-making prostate cancer screening, specifically targeted at African American audiences.	Knowledge; Subjective norms; Behavioural beliefs benefits; Behavioural beliefs barriers and risks; Decision self-efficacy; Screening intention.	16/26

*Included in meta-analysis

#### 3.1.1. Characteristics of disadvantaged groups

Participants fell into one of five disadvantaged groups:

Minority ethnic group (n = 6) [24]–[29];
Low literacy/low education minority ethnic group (n = 6) [30]–[36];
Low literacy group (n = 1) [37];
Low socioeconomic status, including low literacy and/or minority ethnic groups (n = 4) [38]–[41];
Medically underserved (n = 2). [42], [43]

Seven studies [26], [27], [34], [35], [37], [38], [44] included a proportion of participants who were not considered disadvantaged and compared the intervention's effect between disadvantaged participants and those with higher literacy, college education and/or socioeconomic status.

#### 3.1.2. Interventions

The 19 included studies evaluated 21 interventions ranging from communication skills workshops or education sessions [24], [25], [38](n = 4), coaching sessions targeted at patients [43], [45] (n = 2) or health professionals [42] (n = 1), computerised decision aids [26], [30]–[32], [34], [37] (n = 5), video-based interventions to improve informed decision-making and SDM [27], [35], [36] (n = 3),counselling session [39] (n = 1), booklet or DVD decision aids [37], [40], [41] (n = 3) and paper based hand-outs promoting informed decision-making [33] (n = 2).

The design and development of ten out of 21 interventions [24], [25], [27], [28], [33], [34], [37], [39], [40], [46] specifically targeted disadvantaged groups, and focused on literacy, layout, format, and/or cultural differences. Several studies reported the intervention's readability level [35], [41] without specifying whether the intervention had been targeted at disadvantaged groups (e.g. low literacy content).

Seven studies used a theoretical framework in developing the intervention [24], [25], [28], [30]–[32], [34], [41].

#### 3.1.3. Methodological quality

**Figure 2:**
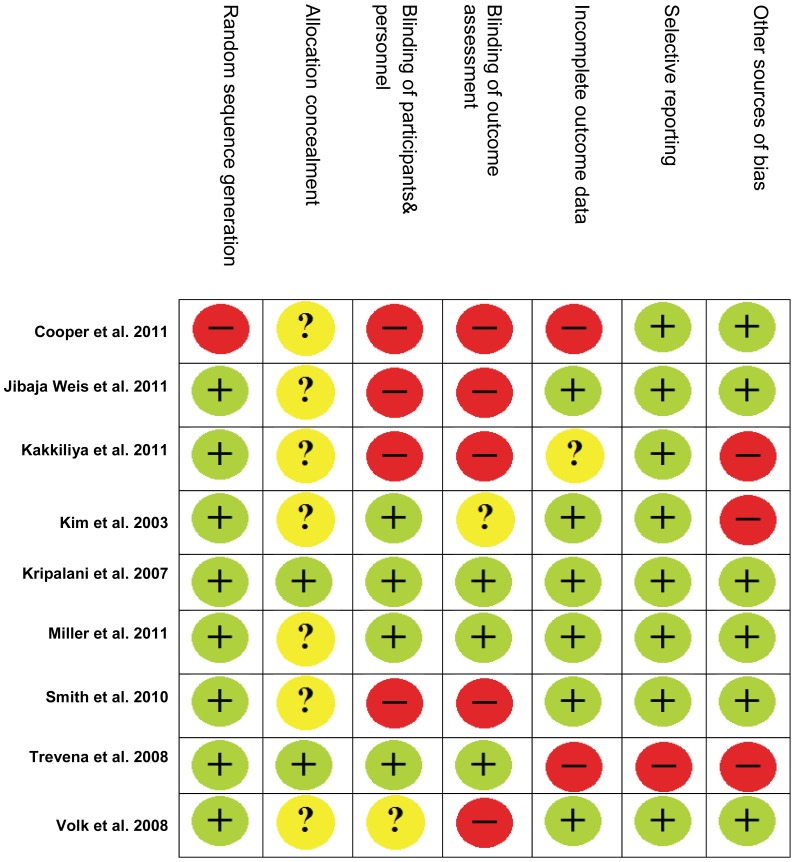
Included studies rated against the Cochrane Risk of Bias tool.

The quality of included studies was low, across both RCTs and observational studies (see Table 1). Observational studies rated against Downs and Black quality assessment checklist ranged from 5 to 16 out of 26. The average score was 11.6. This is low but consistent with Downs and Black's average scores for non-randomised studies (11.7) [20]. Nine studies were rated against the Cochrane risk of bias tool (see Figure 2). Seven out of nine studies did not report allocation concealment while over half of included studies did not blind or clearly report blinding of outcome assessment, participants and personnel.

#### 3.1.4. Data available for meta-analysis

Of 19 studies included in this review, 10 studies had data available and a methodology which permitted inclusion in the meta-analysis [25], [29]–[31], [33], [34], [36]–[38], [40], [41](see Table 1). The remaining studies were excluded as 1) the design and methodology were not compatible with the meta-analytic methodology 2) the published article did not include sufficient information and 3) contacted authors did not provide additional data within stated timescales. We also conducted a narrative synthesis of all included studies.

### 3.2. Meta-analysis

#### 3.2.1. Continuous data

**Figure 3:**
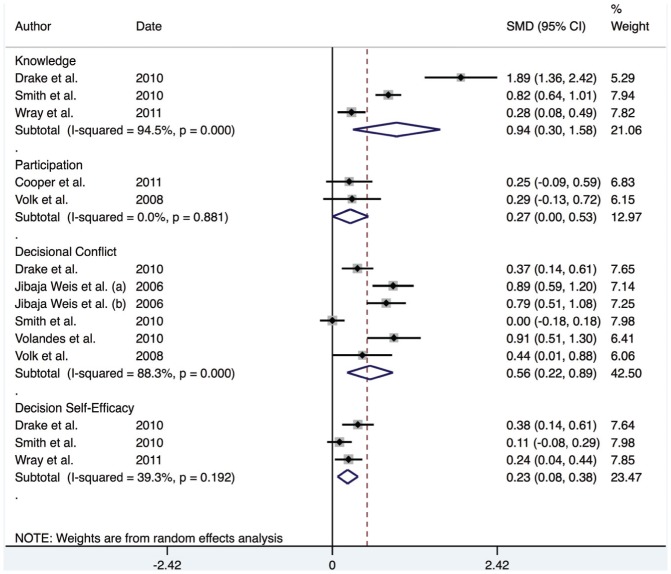
Forest plot for continuous outcomes.

Overall, the meta-analysis for continuous outcomes indicates a moderate positive effect across the domains (knowledge, participation, decisional conflict and self-efficacy), suggesting that the interventions led to an improvement in SDM and increased involvement in healthcare decisions. The reported pooled effect estimates for knowledge, participation, decisional conflict and self-efficacy were 0.94 (95% CI; 0.30–1.58), 0.27 (95% CI; 0.00–0.53), 0.56 (95% CI; 0.22–0.89) and 0.23 (95% CI; 0.22–0.89) respectively (see Figure 3).

Meta-regression analysis was used to identify any covariates that might affect the estimated effect estimates of each domain. The I^2^ was significant for both knowledge and decisional conflict (94.5% and 88.3% respectively) suggesting that a random effects model was suitable. The analysis revealed that use of a theoretical framework, study design, study setting and gender of the study population were not significant predictors of SDM, and did not have an effect on the overall effect estimate (*P*<0.05) across any of the domains.

**Figure 4:**
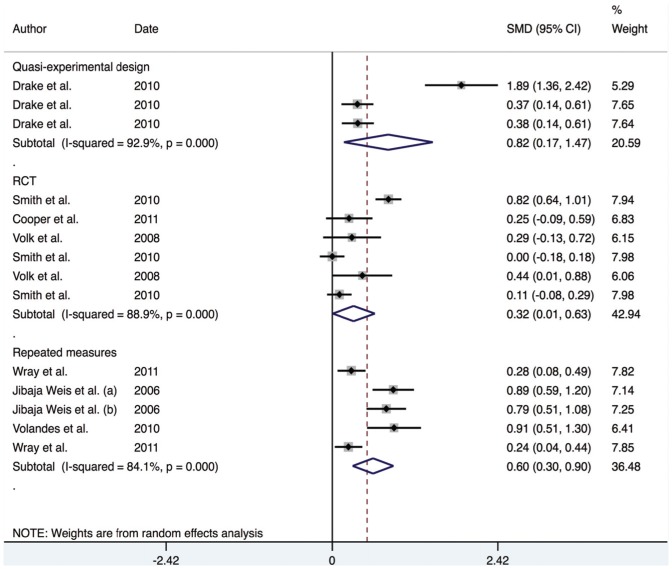
Forest plot for continuous outcomes by study design.

However, given the low number of studies in each group, it is possible that the meta-regression lacked sufficient power to detect the impact of study design on the overall effect estimate. Therefore, an additional analysis, stratified by study design was undertaken. This indicated that the overall effect estimate for RCTs was 0.32 (95% CI; 0.01–0.63), for repeated measures designs was 0.60 (95% CI; 0.30–0.90) and for quasi-experimental designs was 0.82 (95% CI; 0.17–1.47) (See Figure 4). This indicates that the quasi-experimental studies may be inflating the size of the effect compared to RCT and repeated measures designs.

Investigation of the individual studies revealed that Drake's effect estimate for knowledge was a significant outlier compared to the other studies. In order to evaluate the impact of this outlier on the overall effect estimates, a sensitivity analysis was undertaken. The overall effect estimate for the knowledge domain fell from 0.94 to 0.56 with the omission of Drake's study. This also affected the overall effect estimate for the quasi-experimental study design, falling from 0.82 to 0.37. This provided evidence for the large effect that Drake's study was having on the overall effect estimates of both the knowledge domain, and the quasi-experimental designed studies.

**Figure 5:**
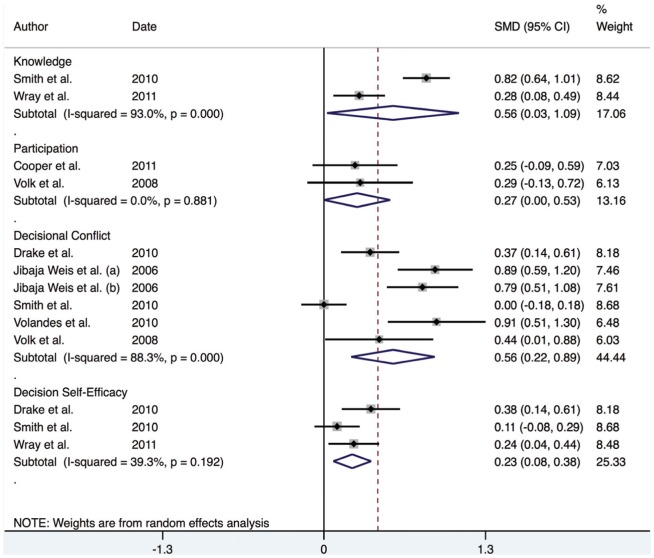
Forest plot for continuous outcomes without Drake study.

Given the evidence that Drake's study investigating knowledge was largely over inflating the effect estimates, a further meta-analysis has been conducted, which has omitted this study (Figure 5).

#### 3.2.2. Binary data

**Figure 6:**
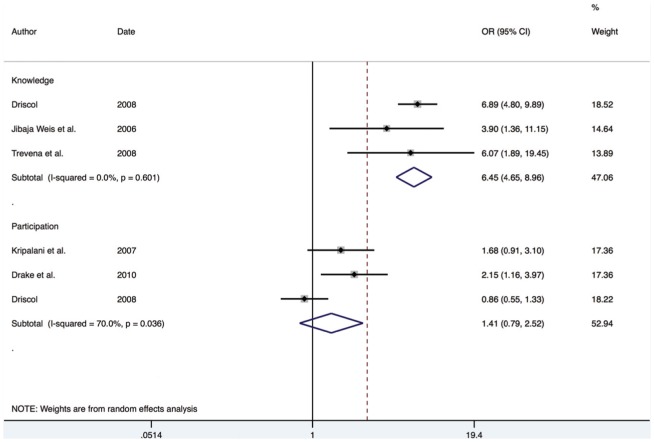
Forest plot for binary outcomes.

The meta-analysis for binary outcomes indicated a large positive effect for knowledge 6.45 (95% CI; 4.85–8.95) and participation 1.41 (95% CI; 0.78–2.52) (see Figure 6).

As is evident from the forest plot, the effect estimate from studies using knowledge outcome measures was markedly larger than those using participation outcome measures. Meta-regression indicated that the type of outcome measure used was having a significant effect on predicting the pooled effect size (*P*<0.05). However, the large interval of the knowledge effect size does raise uncertainty about the precise size of the effect. The I^2^ statistic was significant for the participation domain, suggesting that a random effects model was appropriate. This was likely due to Driscoll's study [38], which indicated a negative impact of SDM.

#### 3.2.3. Publication bias

**Figure 7:**
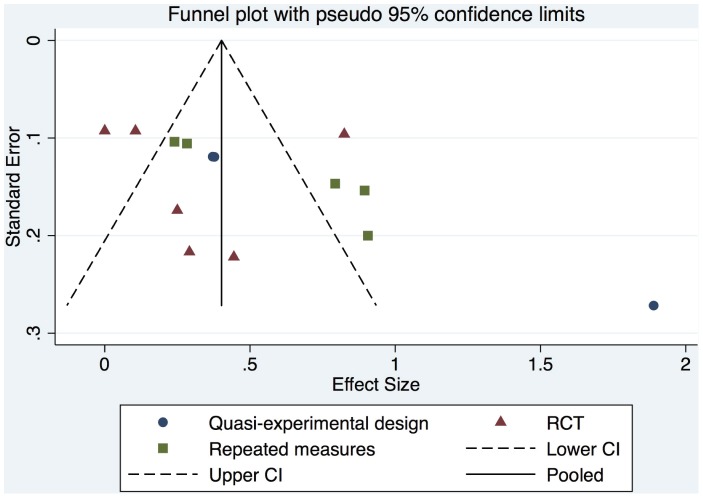
Funnel plot for continuous outcomes.

Funnel plots were used to investigate the potential publication bias of the chosen studies in this meta-analysis. Investigation of the plot (see Figure 7) reveals the large deviation that Drake's study on knowledge was having relative to the other studies, which was investigated in full in the analysis. Otherwise, the plot indicates no obvious gaps, where it would seem that either low or high powered studies where being not published due to either a tendency to report either positive or negative findings. The plot shows a spread of studies at both the high and low end of the sampling range.

### 3.3. Narrative synthesis

#### 3.3.1. Attributes of the decision-making process

Eleven studies assessed the intervention's effect on knowledge, showing significant increases in disadvantaged groups' knowledge levels post intervention, and compared to the control group (when applicable). Further, disadvantaged patients made a significantly more informed choice post-intervention than those in the control group [40], [41] and felt clearer about their values and preferences [32]. Five out of the seven studies that compared disadvantaged groups to higher literacy/education groups measured knowledge post-intervention. Three studies suggested that despite knowledge levels being lower in disadvantaged groups pre-intervention, disparities between groups tended to disappear post-intervention, particularly when the intervention was adapted to disadvantaged groups' needs, e.g. low literacy [35], [37], [38]. However, two studies suggested that lower literacy levels tended to hinder disadvantaged participants' understanding of the intervention's content [26], [27], even when the content had been targeted at mixed literacy groups [27]. Further, the interventions had a significant effect on decisional conflict [30]–[32], [40] (low literacy version of the decisional conflict scale). One study assessed decisional conflict in high and low literacy groups using two intervention types: an ‘edutainment’ (educational entertainment) decision aid intended for low literacy groups and a standard audio-booklet containing the same factual information [37]. Low literacy participants who used the edutainment intervention experienced lower levels of decisional conflict than those who used the standard booklet decision aid. The latter highlights that the format, layout and accessibility of the intervention significantly affected the intervention's impact on disadvantaged patients. There were no statistically significant differences between intervention types among high literacy participants. Further, Volandes et al. assessed decisional conflict in both high and low literacy groups. They demonstrated that uncertainty was higher among low literacy participants pre-intervention but suggested that disparities between groups disappeared post-intervention [36], [44].

Two out of 19 studies showed increased decision self-efficacy scores post intervention among disadvantaged patients [25], [28]. In addition, participants who had used the SDM intervention were likely to prefer a collaborative decision-making style, where the decision was shared with the clinician [25], [40]. Participants were generally more involved in the consultation post-intervention and tended to assume a more active role in discussing options with their clinician [29], [35], [38], [40], [42], [43]. One study found no significant effect of the intervention on confidence in decision-making [40].

There was no statistically significant effect of the SDM interventions on anxiety levels (state and/or trait) [24], [40], [41].

#### 3.3.2. Treatment or screening preferences, intentions and behaviour

Ten studies measured the intervention's effect on treatment or screening preferences, intention and actual behaviour [26], [28], [32]–[34], [36], [38]–[41]. The findings were highly heterogeneous, and it was impossible to identify trends or draw conclusion as to the overall effect of SDM interventions in disadvantaged groups'. Whether or not the intervention affected intention and behaviour was highly dependent on the clinical condition at stake. Taking the example of PSA test, one intervention led to a six fold increase in screening uptake [33] while another increased the proportion of patients feeling undecided [38]. Trevenna et al. found no significant effect of the intervention on screening decisions [41] while Volandes et al. demonstrated a significant change in participants' preferences for treatment post-intervention [36], [44]. Further, Volandes et al. compared preferences for treatment between high and low literacy groups. Pre-intervention, low literacy patients were more likely to prefer aggressive treatments based on a verbal description of dementia, compared to higher literacy groups. These differences disappeared after watching the video intervention.

#### 3.3.3. Adherence and health outcomes

There was no statistically significant effect of the intervention on medication adherence and blood pressure among disadvantaged patients [29]. This study did not compare disadvantaged patients to privileged groups, and did not provide any information on the impact of SDM interventions on adherence levels and condition-specific health outcomes of privileged patients.

#### 3.3.4. Intervention's acceptability

Six studies investigated the intervention's acceptability [24], [27], [30], [31], [37], [40], [41], suggesting that patients were generally satisfied with the content, format, balance between the options portrayed, clarity, length of the intervention, and considered recommending it to others. None of the included studies explicitly evaluated accessibility. Two studies showed that time constraints, combined with the amount of information included in the intervention, tended to limit participants' interaction with the intervention. Participants did not have the time to review all sections, and those in the low-literacy group were occasionally overwhelmed by the amount of information available (particularly in the lower literacy group) [30], [31], [37]. Approximately half of included studies reported using simple language that was tailored to the disadvantaged group's needs. However, across all studies, it was unclear how the readability and accessibility of the intervention had been determined and verified.

#### 3.3.5. Health literacy

Seven studies measured participants' health literacy at baseline [26], [27], [33]–[36], [39], using the Rapid Estimate of Adult Literacy in Medicine (REALM) and the Test of Functional Health Literacy in Adults (TOFHLA). They did not assess the intervention's impact on health literacy. Existing measures of health literacy, such as REALM and TOFHLA, are based on reading skills and comprehension of general health information. Given the SDM interventions included in this review were not designed to improve these basic skills, those interventions were unlikely to have had a direct impact on health literacy, as measured by currently available instruments.

#### 3.3.6. Effect on health inequalities

Seven studies compared the intervention's effect between disadvantaged and higher literacy/higher socio-economic status groups [26], [27], [34]–[38]. Three out of the seven interventions evaluated in those studies [27], [34], [37] had been specifically designed to answer disadvantaged patients' information and decision support needs. Disparities in knowledge, decisional conflict, uncertainty and treatment preferences were narrowed in five out of seven studies, as described above. This suggests that SDM interventions were more beneficial to disadvantaged groups than to privileged participants, and could in turn narrow health disparities.

## Discussion

### 4.1. Main Findings

This review suggests that SDM interventions significantly improved outcomes in disadvantaged groups: increased knowledge, informed choice, participation in decision-making, decision self-efficacy, preference for collaborative decision making and reduced decisional conflict. However, the meta-analysis also indicated that Drake's study was overinflating the overall effect estimates of both the knowledge domain, and the quasi-experimental designed studies. Given the varying quality and designs of the included studies, it is important to interpret these results with caution, and bear in mind the significant effect of Drake's study as an outlier. While inclusion of the Drake study indicated a very large positive effect of SDM on improving patient knowledge, the authors are of the opinion that the effect estimate calculated without the Drake study provides a much more accurate overall effect estimate, indicating only a moderate improvement in knowledge, which certainly fits the pattern seen in the other 3 outcome measures.

The narrative synthesis indicates that disparities in knowledge, decisional conflict, uncertainty and treatment preferences between disadvantaged groups and more privileged populations tended to disappear post-intervention use. Disadvantaged groups may therefore benefit from SDM interventions more than higher literacy/higher education groups. In the long-term, this may reduce health disparities. However, two studies suggested that knowledge gain had been affected by patients' literacy level. This suggests that the intervention's content was perhaps not sufficiently tailored to disadvantaged groups' needs, thus limiting the intervention's impact. The analysis revealed that the layout, use of language, complexity, length and format of the intervention interfered with the intervention's effect among disadvantaged groups, when there were no significant differences among higher literacy patients. In other words, simple and concise interventions, written in plain language and specifically tailored to disadvantaged groups' information and decision support needs appeared most beneficial to underprivileged patients. It is worth noting that 10 out of 21 interventions had been specifically designed to meet the information and decision support needs of disadvantaged patients. It was unclear whether the remaining 11 interventions had taken account of the disadvantaged groups in any way. Finally, these interventions had no significant effect on disadvantaged patients' adherence levels, anxiety, and health outcomes, and no clear effect on screening/treatment preferences, intentions or uptake. This finding is not unique to disadvantaged patients and is consistent with the results of the latest Cochrane review of patient decision aids, which found no clear effect of patient decision aids on adherence, anxiety and condition-specific health outcomes^6^.

### 4.2. Strengths and Weaknesses

Given the paucity of controlled research in this area, we purposefully decided to include all study designs. Limiting the inclusion criteria to randomised controlled trial designs would have more than halved the number of included studies and excluded five out the seven included studies that compared disadvantaged groups with higher literacy/higher education patients. However, this introduced significant heterogeneity, with studies of various designs, and meant that only a proportion of included studies could be pooled in the meta-analysis. In order to account for the heterogeneity, we applied a random effects model to the meta-analysis. In addition, a meta-regression was performed to investigate whether the use of a theoretical framework or the study design were having an impact on the estimation of the overall effect estimate. Furthermore, a stratified analysis was undertaken to investigate how the overall effect estimate varied by study design. This enabled us to see whether certain study designs may have over inflated the overall effect estimate, and could potentially bias the results. Indeed, this was apparent in the investigation of Drake's study, which was found to be over-inflating the effect estimate for knowledge.

Further, the quality of included studies was variable, and fairly low. However, this is consistent with the quality assessment scores reported in the Cochrane review of Decision Aids for people facing health treatments or screening decisions and highlights the need for further improvement in the methodological quality of observational and interventional studies assessing the impact of shared decision-making interventions [6]. Publication and other selection biases are a potential threat to validity in all systematic reviews and we cannot exclude the possibility that some studies were missed resulting in reduced precision and the potential for bias. However, we made considerable efforts to identity all eligible studies, published and unpublished by searching the grey literature, conference proceedings, using a “cited by” search and “related articles” search in PubMed and by contacting experts in this area through social media. We also used funnel plots to investigate potential publication biases. Nonetheless, since we are not combining all the outcomes of the included studies and had to stratify them, the number of studies available for investigation was limited. The funnel plots showed that there was a lack of studies with a high number of participants.

In addition, the sample size was generally small and follow-up was not systematic and limited. It is therefore difficult to infer whether the impact of these interventions would last beyond funded research and could reduce health inequalities, long term, in routine care. Only seven studies compared the interventions' effect between disadvantaged groups and higher literacy/higher socioeconomic status populations. Although a trend indicated that SDM interventions may benefit disadvantaged groups more than higher literacy groups, these findings were not pooled in the meta-analysis and should therefore be interpreted with caution.

Finally, as previously stated, 10 out of the 21 interventions included in this systematic review had been specifically designed with disadvantaged patients' needs in mind. This is primarily due to the fact that our search specifically targeted disadvantaged groups and therefore included studies that had focussed on this group. This may therefore have increased the likelihood that the interventions would benefit disadvantaged groups, and is unlikely to be representative of interventions commonly available to all patients in routine clinical settings worldwide.

### 4.3. Comparison with other studies

Disadvantaged groups experience an unfair paradox. They are at increased risk of poorer health outcomes and heavier disease burden than the rest of the population [47]–[49]. Nonetheless, they struggle to access relevant services, to use and understand health information and to actively engage in healthcare. Difficulties range from booking appointments, understanding general health information, consent forms, medication regimens, to advocating for their health and discussing their options and preferences with health professionals. This group has most to gain from engaging in healthcare but is currently disproportionately burdened and marginalised. King et al. advance that existing informed consent procedures are unfit for lower literacy and underprivileged patients, failing to inform and engage them to a satisfactory level. They advocate for the potential of SDM interventions to shift this balance [50]. McCaffery et al. propose a framework to tailor SDM related interventions and developments to the needs of those with lower literacy, knowledge and confidence [51]. They argue that using plain language information, avoiding complex medical jargon and avoiding the passive voice, will make significant improvements to lower literacy groups' understanding and activation levels. They describe simple layout, design, graphic display formats, and accessibility principles, which, if systematically applied, could directly benefit marginalised patients [52]. The authors advocate that patients' ability to engage in, and benefit from SDM largely depends on their capacity to understand and use the tools available to them.

As illustrated above and in the present review, the potential for SDM interventions to reduce health inequalities and engage disadvantaged patients will essentially be realised if tools and processes are tailored to their needs. Although a significant proportion of the interventions included in this review were specifically targeted to disadvantaged groups' needs, this is not yet the norm in clinical settings. The majority of available interventions worldwide remain information-heavy, web-based, sophisticated and requiring high levels of health and computer literacy [10]. Existing programmes and interventions introduce the risk that health inequalities may prevail, or even increase, if current techniques and interventions remain unadapted to those who need it most. Similarly, it is highly likely that in contexts where SDM is not actively promoted and supported by a trained clinician and/or an intervention, disadvantaged patients are most likely to be marginalised, therefore increasing health inequalities. However, this important question is beyond the remit of this review (which exclusively focused on the impact of SDM interventions) and requires further investigation. In the present review, Volk et al. emphasised that the edutainment decision aid, although beneficial in disadvantaged groups, was deemed to contain too much information. Shorter tools, which only provide essential information and can be written in simpler language may be more adapted [53]. Further, the majority of existing interventions are web-based. This presupposes access to a computer, computer literacy and may introduce skill barriers, which in turn, may exclude underprivileged groups [54]. Finally, adapting tools to suit disadvantaged groups' needs does not solely rest on writing simple, accessible content in plain language. Polaceck et al. postulate that breast cancer disparities in the US may be reduced if women are provided with culturally and ethnically appropriate professional support and information [55].

### 4.4. Conclusions

Promoting SDM in clinical settings is an ethical imperative for all clinicians and a priority on the policy agenda. This review demonstrates the beneficial impact of SDM interventions on disadvantaged groups, across various outcomes, and highlights the potential for SDM and related interventions to reduce health inequalities when the intervention is adapted to disadvantaged groups' needs. It is therefore essential to support groups who are burdened by worse health outcomes and traditionally disengaged, by tailoring communication, information and SDM interventions to their specific needs: using plain language information, avoiding complex medical jargon and using shorter interventions, with simpler layouts and formats. Although interventions will play an important part in increasing patient participation in healthcare, the role of health professionals in supporting disadvantaged patients and tailoring information to their needs is essential [51], [55], [56]. Clinicians should see SDM as an opportunity to include and empower those who are normally disengaged by using tools and processes that are simple and sufficiently accessible to benefit all groups, and particularly those who are traditionally marginalised.

## Supporting Information

Protocol S1 **Systematic review protocol.**
(DOC)

Checklist S1 **PRISMA checklist.**
(DOC)
