# Supplementary material for: Do Interventions Designed to Support Shared Decision-Making Reduce Health Inequalities? A Systematic Review and Meta-Analysis
Source: PLoS One. 2014 Apr 15;9(4):e94670. doi: 10.1371/journal.pone.0094670 (PMC3988077; doi:10.1371/journal.pone.0094670)
Supplement: Protocol S1 — Systematic review protocol. (DOC) [file pone.0094670.s001.doc]

Systematic Review Protocol

Does shared decision-making reduce health inequalities: a systematic review

1. Background

Shared Decision-making: A Definition

Shared Decision-making offers a new paradigm to manage patients’ growing demand for healthcare by promoting patient involvement in medical decision-making and underpins the Department of Health’s vision of a patient-centred NHS: “No decision about me without me” (NHS White Paper 2010). It is a process in which patients become involved as active partners with their clinicians, to clarify acceptable options and choose their preferred course of care, one that is ideally aligned with their values and preferences. Shared Decision-making assumes informed and empowered patients who have developed the skills, knowledge and confidence required to discuss their options with clinical experts, and influence their care and clinical outcomes. Shared Decision-making is especially relevant in situations of clinical equipoise, where one specific course of action cannot benefit all and available options involve significant harms and benefits (Godlee 2005). The patient’s values, lifestyle and preferences may therefore be determinant in choosing a preferred course of care, and warrant participation in medical decision-making.

Benefits of Decision Support Interventions

Interventions, known as patient decision Aids or Decision Support Interventions, have been developed to help patients clarify their values and preferences while providing evidence-based information about the options’ harms, benefits and outcome probabilities (Estabrooks et al. 2001; Molenaar et al. 2000; O'Connor et al. 1999). According to the Cochrane systematic review of decision aids, “Decision aids differ from usual health education materials because of their detailed, specific, and personalised focus on options and outcomes for the purpose of preparing people for decision-making” (O'Connor et al. 2006, p. 2). The past 10 years have seen a proliferation of patient decision aids addressing over 23 clinical decisions in a variety of formats (paper, audio, video, web-based)(O'Connor et al. 2006). In 2006, it was estimated that these interventions were accessed about 9 million times, principally online (O'Connor et al. 2007). The latest Cochrane review of Patient Decision Aids demonstrated the following benefits: increased knowledge, more realistic expectations, increased participation in decision-making and reduced decisional conflict and indecision compared to usual practice (Stacey et al. 2011). Although the evidence remains scarce, other likely benefits of Shared Decision-making include reductions in the uptake of elective surgical procedures, unwarranted practice variation and litigation costs (Stacey et al. 2011). It has also been hypothesised that Shared Decision-making would reduce health inequalities by reaching and empowering socioeconomic groups who are more vulnerable and traditionally less engaged, therefore narrowing health inequalities (Stacey et al. 2011; Coulter & Collins 2011). Academics, policy-makers and developers of Decision Support Interventions have traditionally believed that Shared Decision-making and related interventions would benefit all groups, and especially underprivileged and lower literacy groups, by increasing their involvement in healthcare decisions, fostering informed choice, and in turn, improving health outcomes. The latest King’s Fund report on Shared Decision-making postulates that “people from disadvantaged groups have most to gain”. However, the evidence behind this assumption is poor as only few studies to date have assessed the impact of shared decision-making on disadvantaged and lower literacy populations, and on health inequalities overall. The Cochrane systematic review of patient decision aids did not assess the impact of patient decision aids on disadvantaged groups and health inequalities and called for more research to ensure that existing and future interventions were accessible and beneficial to traditionally less engaged and underprivileged populations. While the link between Shared Decision-making and health inequalities has not yet been established, there is documented evidence that specific patient groups are more likely to become “activated” and engaged in decision-making (Thomson et al. 2005). Research shows that younger patients, women and people from higher socioeconomic groups are more likely to engage in healthcare decisions, and assume an active role in medical decision-making than men (Say et al. 2006; Leon et al. 2001; Thomson et al. 2005).

Why it is important to do this review

However, very few studies and no systematic review to date, have investigated the impact of Shared Decision-making interventions on disadvantaged groups and health inequalities.

2. Aims

The aim of this review is to assess the impact of Shared Decision-making interventions on patients from disadvantaged groups, and infer the effect of shared decision-making on health inequalities.

The secondary or practical aim of this review is to assess the quality and accessibility of existing Shared Decision-making interventions, in order to identify the features that improve outcomes for disadvantaged groups, and potentially reduce health inequalities.

3. Research questions

- Can Shared Decision-making interventions improve outcomes for disadvantaged groups?
- What are the features of Shared Decision-making interventions that are beneficial to disadvantaged groups and influence health inequalities?
- Can Shared Decision-making and related interventions reduce health inequalities?

4. Study selection criteria

**4.1 Types of studies**

All observational, interventional as well as qualitative studies (published in all languages) will be included if they meet at least one of the following criteria: (1) assess the effect of shared decision-making interventions on disadvantaged groups and/or health inequalities or include at least 50% of people from disadvantaged groups or report results for that group separately; 2) compare the effect of shared decision-making interventions between disadvantaged groups and people from higher socioeconomic groups and literacy levels, 3) measure the effect of a shared decision-making intervention on literacy and/or health inequalities using a valid instrument, (4) assess the quality and/or accessibility of Shared Decision-making interventions specifically designed for disadvantaged groups or used by disadvantaged populations.

**4.2 Types of participants**

Participants aged 18 or over from disadvantaged groups who are facing a ‘preference-sensitive’ healthcare decision. Disadvantaged group is defined as all people who are socially disadvantaged in respect of: 1) poverty/socioeconomic status; 2) ethnic minority status; 3) education/literacy level or 4) geographical location (areas described as disadvantaged/or medically underserved). Studies of psychiatric patients will be included. All conditions and clinical settings (e.g. lay care, primary, secondary care) will be included.

Studies that have not described their cohort as one that includes disadvantaged groups will be included as long as the outcomes include a valid measure of literacy or numeracy or reading ability or health inequality.

**4.3 Types of interventions**

We will include all interventions designed to engage disadvantaged patients in medical decision-making and/or facilitate shared decision-making, patient involvement and patient activation. This includes decision support interventions (also known as Patient Decision Aids), decision coaching, and education packages as long as the intervention also aims (in addition to informing and educating patients) to involve patients in decision-making and help patients clarify their values and preferences.

We are particularly interested in interventions that have been specifically designed for use by disadvantaged groups. However, we will include all interventions as long as their aim is to engage patients in medical decision-making, promote shared decision-making, promote patient involvement, patient activation and elicit patient values and preferences. All formats of interventions will be included: web-based, video, audio and printed materials.

**4.4 Types of outcome measures**

We will include all outcome measures, with a specific focus on measures of health literacy and health inequality.

The **primary outcomes measures** will be:

- Health literacy, including general literacy skills, numeracy skills and reading ability;
- Health inequality.

The **secondary outcome measures** will include all other outcome measurements such as, for example:

- Attributes of the choice: knowledge, accurate risk perceptions, value congruence with chosen option;
- Attributes of the decision-making process: decisional conflict, patient-practitioner communication, participation in decision-making, proportion undecided, satisfaction;
- Behaviour: choice (actual choice implemented, option preferred or intention as surrogate measure);
- Health outcomes: health status, quality of life, anxiety, depression, emotional distress, regret, confidence;
- Accessibility and acceptability of the intervention.

5. Search strategy

Electronic searches will be performed on Medline, Embase, PsychINFO, CINAHL, Web of Science, the Database of Abstracts of Reviews of Effectiveness (DARE), and the Cochrane Library from inception until May 2012.

- The reference list of all primary and review articles will be searched manually to identify studies which have not been picked-up by the electronic search. A citation search will also be performed using the ‘cited by’ option on web of Science, Google Scholar and Scopus, and the ‘related articles’ option on PubMed and Web of Science.
- Key Journals, grey literature (i.e. technical reports, work in progress), conference proceedings (international Conference on Shared Decision-making and conferences of the Society for Medical Decision-making), and the National Center for the Study of Adult Learning and Literacy (until 31 July 2007) will also be searched.
- Research registers.
- The Internet.

A list of key words and subject headings (MeSH words in PubMed) will be written in Ovid Medline and run in each database (see appendix 2).

5.1 Preliminary search

A preliminary search was conducted in Ovid Medline to identify existing systematic reviews and assess the volume of potentially included articles. No systematic review specifically investigating the relationship between Shared Decision-making and health inequalities was identified. The search retrieved several reviews assessing patient preferences for shared decision-making in a variety of clinical contexts, without specifically investigating the impact of SDM on health disparities.

**5.2 Data extraction and analysis**

An independent double data extraction will be performed, using a pre-designed form, adapted from the Cochrane Effective Practice and Organisation of Care (EPOC) collection checklist (EPOC 2008). Inconsistencies will be resolved by discussion. In the first stage of the review, we will assess the titles and abstracts of all articles retrieved by electronic searches. A full-text analysis of all citations meeting the selection criteria will then be conducted.

We will extract information about 1) the author(s), 2) publication year, 3) country, 4) type of study design, 5) aim(s) and research questions, 6) type of participants and sample size, 7) data collection method (i.e. measure of Shared Decision-making/patient activation/patient involvement), 8) response rate, 9) method(s) of analysis, 10) outcomes.

Information about the accessibility of the interventions will also be collected.

Additional information about the duration of follow-up, characteristics of the Shared Decision-making intervention and control groups, as well as key findings, will be extracted from the interventional studies.

A narrative review will be produced independently of the heterogeneity of included studies.

In parallel, heterogeneity will be assessed using the Chi-Square test and I^2^ test (Higgins 2002). If there is sufficient homogeneity, studies will be pooled in a meta-analysis with dichotomous outcomes presented as relative risks (RR) and continuous data as mean differences (MD). However, in the likely event of heterogeneity studies will not be pooled but instead we will present data narratively with an indication of whether the effect of the intervention was positive, negative or not statistically significant. Where possible we will report dichotomous outcomes as relative risks and continuous data as mean differences, both with 95% confidence intervals.

5.3 Study quality assessment

In addition to the above selection criteria, the quality of all included studies will be considered and appraised. The quality assessment will be informed by the Cochrane risk of bias tool, the PRISMA statements of standards for assessing systematic reviews (Moher et al. 2009), as well as the Jadad and CONSORT scales for RCTs (Jadad et al. 1996; Moher et al. 2001). The quality of qualitative studies will be assessed using Spencer’s framework (Spencer et al. 2003). The resulting checklist will be used by two independent assessors against all included studies. Discrepancies will be resolved by discussion and consensus.

References

Coulter, A., Collins, A. Making shared decision-making a reality - No decision about me without me. The King’s Fund 2011.

Department of Health. Equity and excellence: liberating the NHS. NHS White Paper. 12 July 2010.

EPOC 2008. Cochrane Effective Practice and Organisation of Care Review Group. Data collection checklist. http:// www.epoc.cochrane.org [Last access date: 2008–12–03].

Estabrooks, C. et al. 2001. Decision aids: are they worth it? A systematic review. Journal of Health Services Research and Policy 6(3), pp. 170-182.

Godlee, F. 2005. Clinical Evidence. BMJ Publishing Group.

Jadad AR, Moore RA, Carroll D, et al. Assessing the quality of reports of randomized clinical trials: is blinding necessary? Control Clin Trials 1996;17:1e12.

Higgins JG, S (editors). Cochrane Handbook for Systematic Reviews of Interventions Version 5.0.1 [updated September 2008]. The Cochrane Collaboration, 2008. Available from [www.cochrane-handbook.org](http://www.cochrane-handbook.org/) 2008.

Higgins JP, Thompson SG. Quantifying heterogeneity in a meta-analysis. Stat Med. 2002 Jun 15;21(11):1539-58.

Leon D.A., Walt G., Satariano W.A. Poverty, inequality and health: an international perspective. Am J Epidemiol 2001;154:588–9.

Moher D, Schulz KF, Altman DG. The CONSORT statement: revised recommendations for improving the quality of reports of parallel-group randomised trials. Lancet 2001; 357:1191–4.

Moher D, Liberati A, Tetzlaff J, Altman DG. Preferred reporting items for systematic reviews and meta-analyses: the PRISMA statement. BMJ 2009; 339:b2535.

Molenaar, S. et al. 2000. Feasibility and effects of decision aids. Medical Decision-making 20(1), pp. 112-127.

O'Connor, A.M. et al. 1999a. Decision aids for patients facing health treatment or screening decisions: systematic review. British Medical Journal 319(7212), pp. 731-734.

O'Connor, A.M. et al. 2006. Decision aids for people facing health treatment or screening decisions. The Cochrane Library 4, pp. 1-110.

[Say R](http://www.ncbi.nlm.nih.gov/pubmed?term="Say R"%5BAuthor%5D), [Murtagh M](http://www.ncbi.nlm.nih.gov/pubmed?term="Murtagh M"%5BAuthor%5D), [Thomson R](http://www.ncbi.nlm.nih.gov/pubmed?term="Thomson R"%5BAuthor%5D). Patients' preference for involvement in medical decision-making: a narrative review. [Patient Educ Couns.](http://www.ncbi.nlm.nih.gov/pubmed/16442453) 2006 Feb;60(2):102-14.

Spencer, L., et al. (2003). *Quality in Qualitative Evaluation: A Framework for Assessing Research Evidence*. 2003, Cabinet Office. Available from www.gsr.gov.uk/downloads/evaluating_policy/a_quality_framework.pdf: London (UK): Government Chief Social Researcher's Office.

[Stacey D](http://www.ncbi.nlm.nih.gov/pubmed?term="Stacey D"%5BAuthor%5D), [Bennett CL](http://www.ncbi.nlm.nih.gov/pubmed?term="Bennett CL"%5BAuthor%5D), [Barry MJ](http://www.ncbi.nlm.nih.gov/pubmed?term="Barry MJ"%5BAuthor%5D), [Col NF](http://www.ncbi.nlm.nih.gov/pubmed?term="Col NF"%5BAuthor%5D), [Eden KB](http://www.ncbi.nlm.nih.gov/pubmed?term="Eden KB"%5BAuthor%5D), [Holmes-Rovner M](http://www.ncbi.nlm.nih.gov/pubmed?term="Holmes-Rovner M"%5BAuthor%5D), [Llewellyn-Thomas H](http://www.ncbi.nlm.nih.gov/pubmed?term="Llewellyn-Thomas H"%5BAuthor%5D), [Lyddiatt A](http://www.ncbi.nlm.nih.gov/pubmed?term="Lyddiatt A"%5BAuthor%5D), [Légaré F](http://www.ncbi.nlm.nih.gov/pubmed?term="Légaré F"%5BAuthor%5D), [Thomson R](http://www.ncbi.nlm.nih.gov/pubmed?term="Thomson R"%5BAuthor%5D). Decision aids for people facing health treatment or screening decisions. [Cochrane Database Syst Rev.](http://www.ncbi.nlm.nih.gov/pubmed/21975733) 2011 Oct 5;(10):CD001431.

Thomson R, Murtagh M, Khaw FM. Tensions in public health policy: patient engagement, evidence-based public health and health inequalities. Qual Saf Health Care 2005;14:398–400.

**Appendix 1**

# Search methods for identification of studies

## Electronic searches

We will perform a comprehensive literature search to identify relevant studies in the following electronic databases (Table 1). A search strategy will be developed using a comprehensive list of keywords and MeSH headings. The search strategy will be tested for key papers on the OVID Medline database 1946-2012 (See Appendix 1). The search strategy will be modified to search rest of the bibliographic databases. There will be no restrictions based on language, date or publication status

Table 1.

| CINAHL (*Cumulative Index to Nursing and Allied Health Literature)* | 1982 – 2012 |
| --- | --- |
| Cochrane Central Register of Controlled Trials | 1996 – 2012 |
| Cochrane Database of Systematic Reviews (CDSR), | 1996-2012 |
| EMBASE | 1980 -2012 |
| HMIC (*Health Management Information Centre*) | 1979-2012 |
| MEDLINE | 1946 – 2012 |
| MEDLINE In-Process and Other Non-Indexed Citations | 1951-2012 |
| NHSEED (*NHS Economic Evaluation Database*) | 1979 – 2012 |
| Open SIGLE (System for Information on Grey Literature in Europe) | 1980 - 2005^*^ |
| Psycinfo | 1806-2012 |
| Web of Knowledge — ISI Proceedings | 1990 - 2010 |
| Web of Knowledge — ISI Science Citation Index | 1981 – 2012 |
| Web of Knowledge — ISI Social Science Citation Index | 1981 - 2012 |

## Searching other resources

| **Websites** |
| --- |
| Google Scholar search <http://scholar.google.co.uk/> |
|  |
| **Journals** |
|  |
|  |
|  |
|  |
|  |

The search strategy will be modified to search rest of the bibliographic databases. In addition, a range of ‘snowballing*’* techniques will be used to increase the sensitivity of the search including reference list follow up, contact with subject experts and relevant websites/organisations, and table of content scanning for the top two to three most frequently cited journals.

**Search Strategy - OVID Medline 1946- June 2012**

1. exp Decision-making/

2. Physician-Patient Relations/

3. Patient Participation/

4. physician-patient relation*.mp.

5. doctor-patient relationship*.mp.

6. patient decision-making.mp.

7. share* decision mak*.mp.

8. shared decision-making.mp.

9. ((decis* or information) adj3 (choic* or aid*)).mp.

10. informed decision*.mp.

11. (decision support adj (strat* or method*or technique* or intervention*)).mp.

12. making decision*.mp.

13. Health communication/

14. *Communication/

15. or/1-14

16. Healthcare Disparities/

17. Educational Status/

18. Socioeconomic Factors/

19. Health Status/

20. Health Status Disparities/

21. health inequalit*.mp.

22. vulnerable populations.mp.

23. medically underserved area.mp.

24. underserved populations.mp.

25. Minority Groups.mp.

26. ethnic groups.mp.

27. health inequalit*.mp.

28. health disparit*.mp.

29. (educational status or health status).mp.

30. socioeconomic factors.mp.

31. or/16-30

32. Decision aid*.mp.

33. decision support method*.mp.

34. decision-making technique*.mp.

35. exp Decision Support Techniques/

36. Decision-making, Computer-Assisted/

37. Decision Support Systems, Clinical/

38. patient education.mp.

39. Patient Education as Topic/

40. Health Education/

41. (decision adj3 (aids or strat* or method*or technique* or intervention*)).mp.

42. (decision support adj (strat* or method*or technique* or intervention*)).mp.

43. (decision-making adj3 (programme* or approach*)).mp.

44. Educational Technology/

45. or/32-44

46. Information Literacy/

47. low literacy.mp.

48. health literacy.mp.

49. literacy.mp.

50. Computer Literacy/

51. numeracy.mp.

52. wide range achievement test.mp.

53. (Rapid estimate adj2 adult literacy).mp.

54. (functional health literacy adj2 adults).mp.

55. reading abilit*.mp.

56. reading skill.mp.

57. or/46-56

58. 31 or 57

59. 15 and 45 and 58
